# Efficacy analysis of targeted nanodrug for non-small cell lung cancer therapy

**DOI:** 10.3389/fbioe.2022.1068699

**Published:** 2022-11-08

**Authors:** Tongtong Li, Tong Zhou, Ying Liu, Jingyue Wang, Zhenxiang Yu

**Affiliations:** ^1^ Department of Respiratory Medicine, The First Hospital of Jilin University, Changchun, China; ^2^ Department of Endocrinology and Metabolism, The First Hospital of Jilin University, Changchun, China; ^3^ Department of Cardiology, The First Hospital of Jilin University, Changchun, China

**Keywords:** non-small cell lung cancer, antitumor efficacy, nanoparticles, RGD peptide, vincristine

## Abstract

Biological macromolecules have been widely used as biomedical carriers in treating non-small cell lung cancer (NSCLC) due to their biocompatibility, targeting, biodegradability, and antitumor efficacy. Nanotechnology has been used in clinics to treat many diseases, including cancer. Nanoparticles (NPs) can accumulate drugs into tumors because of their enhanced permeability and retention (EPR) effects. However, the lack of active targeting ligands affects NPs drug delivery. Arginine-glycine-aspartic (RGD), as a targeting ligand, has distinct advantages in targeting and safety. In the present study, an RGD peptide-modified nanogel called RGD−polyethylene glycol−poly (L-phenylalanine-*co*-L-cystine) (RGD−PEG−P (LP-*co*-LC−P (LP-*co*-LC) was investigated to deliver vincristine (VCR) as NSCLC therapy. The VCR-loaded targeted nanoparticle (RGD-NP/VCR) demonstrated excellent antitumor efficacy compared to the free drug (VCR) and untargeted nanoparticle (NP/VCR) without any significant side effects. RGD-NP/VCR has better tumor inhibition and fewer side effects, indicating its potential benefit in NSCLC treatment.

## 1 Introduction

Lung cancer, with the highest incidence, has become the leading cause of cancer-associated death worldwide, with NSCLC accounting for 80%–85% of all cases (Cheng Y. et al., 2021; Pantazaka et al., 2021). The NSCLC treatment options include surgery, targeted therapy, chemotherapy, radiotherapy, and immunotherapy (Qu et al., 2020). Many lung cancer patients miss surgery because they are diagnosed in advanced stages. The basic treatment of NSCLC is chemotherapy, which can be used as adjunctive therapy for early resectable lung cancer and palliative therapy for advanced lung cancer (Pirker, 2020). The development of targeted therapy and immunotherapy have altered the therapeutic pattern and prognosis of NSCLC. However, as therapeutic selectivity increases, drug efficacy and applications are constrained by drug resistance and toxic side effects (Meador and Hata, 2020).

Chemotherapy has a good efficacy as a conventional therapy but has a low long-term response rate due to a lack of tissue specificity and high off-target toxicity (Garcia-Fernandez et al., 2020). The primary issues with chemotherapy include cytotoxicity and drug resistance. VCR is an anticancer drug derived from the periwinkle plant that can affect mitosis by inhibiting tubulin binding and is used to treat various cancers (Skubnik et al., 2020; Skubnik et al., 2021). Although it has significant antitumor efficacy, it has been associated with high incidence of side effects due to its lack of cell selectivity. VCR can cause alopecia, nausea, vomiting, peripheral neuropathy, and other side effects, the most serious of which is peripheral neuropathy (Skubnik et al., 2021). Peripheral neuropathy has a significant impact on a patient’s quality of life. Therefore, its clinical applications are limited and better suited for combination therapy (Verma et al., 2020). Thus, it is critical to investigate new approaches for reducing VCR side effects.

Nanodrugs have increasingly been used in tumor screening, diagnosis, and treatment in recent years (Karpuz et al., 2020). NPs include polymer, biological, liposome, and metal NPs (Mukherjee et al., 2019). NPs can be secreted in blood vessels surrounding tumors and accumulate in tumor cells, a passive process known as the EPR effect (Shi et al., 2017; Nam et al., 2019; Ezhilarasan et al., 2022). However, the unstable nature or easy dissociation of these nanodrugs in the biological environment results in reduced targeting and therapeutic efficiency (Ouyang et al., 2022). Therefore, it is imperative to develop a stable nanomaterial to improve targeting efficiency.

The RGD sequence, whose receptor belongs to the integrin family, was discovered 30 years ago (Sheikh et al., 2022). Integrins can regulate cell proliferation, migration, tumorigenesis, and metastasis. It can also transmit signals to the interior of the cell (Cooper and Giancotti, 2019). It was identified that integrin avβ3 plays a positive role in tumorigenesis with increased expression in a variety of tumor cells, making it a potential target for tumor therapy (Guo and Giancotti, 2004; Desgrosellier and Cheresh, 2010; Cooper and Giancotti, 2019; Xiao et al., 2019). RGD peptide can bind to integrin avβ3 and is frequently used as targeting ligands (Yu et al., 2014; Mohebbi et al., 2019). RGD peptide-modified NPs have a high affinity for integrin overexpressed tumor cells and can improve the antitumor efficacy by inhibiting integrin function and enhancing targeting. (Zhang et al., 2011; Ahmad et al., 2021).

In the present study, RGD-NP/VCR was explored for NSCLC therapy. The findings revealed that RGD-NP/VCR had superior antitumor efficacy and fewer side effects. It has been demonstrated that RGD-modified NPs are an effective drug delivery system (DDS) that can improve targeting and antitumor efficacy.

## 2 Materials and methods

### 2.1 Materials

Lewis lung cancer (LLC) cell lines were bought from Kang Lang Biotechnology Co., LTD. (Shanghai, PR China). Dulbecco’s modified eagle medium (DMEM) was purchased from Gibco (Gland Island, NY, United States). The antibody of Caspase-3, Bax, Ki-67, and Bcl-2 were obtained from Abcam Company (Cambridge, United Kingdom). Streptomycin and penicillin were supplied by North China Pharmaceutical Co. LTD., Shijiazhuang, China. 4′,6-diamidino-2-phenyl**i**ndole (DAPI) and 3-(4,5)-dimethylthiahiazo (-z-y1)-3,5-di- phenytetrazoliumromide (MTT) were purchased from Sigma-Aldrich (Shanghai, P. R. China). TUNEL kit was purchased from Sigma-Aldrich (United States). BALB/c mice (5-week-old) weighing 18.0 ± 3.2 g were obtained from the Charles River Laboratories of Beijing. All mouse-related experiments were performed in accordance with the Animal Care and Use guidelines of Jilin University.

### 2.2 Preparation and characterization of RGD−polyethylene glycol−poly (L-phenylalanine-*co*-L-cystine) and methoxy polyethylene glycol−poly (L-phenylalanine-*co*-L-cystine)

The RGD peptide-modified nanogel called RGD−polyethylene glycol−poly (L-phenylalanine-*co*-L-cystine) (RGD−PEG−P (LP-*co*-LC; RGD-NP) and untargeted nanogel called methoxy polyethylene glycol−poly (L-phenylalanine-*co*-L-cystine) (mPEG−P (LP-*co*-LC; NP) were prepared by previous method (Li et al., 2018; Ding et al., 2022), which were provided from the Changchun Institute of Applied Chemistry, Chinese Academy of Sciences. VCR. 20.0 mg of mPEG−P (LP-*co*-LC) or RGD−PEG−P (LP-*co*-LC) was first dispersed in 5.0 ml of N,N-dimethylformamide, and then 5.0 mg of vincristine (VCR) was dissolved in the above solution and further stirred for 2 h. Subsequently, 5.0 ml of phosphate buffered saline (PBS) was dropwise added into the above mixture. Finally, the NP/VCR and RGD-NP/VCR were obtained after dialyzed and lyophilization. Transmission electron microscope (TEM) measurements was performed on a JEOL JEM-1011 TEM (Tokyo, Japan). Dynamic laser scattering (DLS) measurements of nanoparticles was performed with a vertically polarized He−Ne laser (DAWN EOS; Wyatt Technology, Santa Barbara, CA, United States).

### 2.3 Cell culture

LLC cells were cultured in completed DMEM supplemented with 10% fetal bovine serum (FBS), streptomycin (50.0 IU ml^−1^) and penicillin (50.0 IU ml^−1^) before being placed in a 37°C, 5% CO2 incubator. The culture medium was changed every 1–2 days, and the passage was digested with 0.25% trypsin every 3 days.

### 2.4 MTT assay

MTT assay was used to determine the cytotoxicity of VCR, NP/VCR, and RGD-NP/VCR. LLC cells were seeded in 96-well plates at a density of 1 × 10^4^ cells per well and incubated for 48 h. The VCR, NP/VCR, and RGD-NP/VCR were added in culture medium in different concentrations including 1.00, 0.50, 0.25, 0.13, 0.06, 0.03, 0.02, and 0.01 μg ml^−1^, respectively. After 48 h incubation, the 10.0 μL stock solution containing 5 mg ml^−1^ MTT was added to each wall and incubated further for 4 h. In each well, 150 μL dimethyl sulfoxide (DMSO) was added and shaken for 5 min at 1,440 rpm. The experiment was triplicate, and the optical density (OD) was measured at 492 nm using a microplate reader. A nonlinear regression analysis was used to calculate the IC_50_s (half maximal inhibitory concentrations) of three drugs. Cell viability was measured by Eq. 1:

$$Cell\;viability\;\left(\%\right)=\frac{The\;absorbance\;of\;the\;experimental\;group}{The\;absorbance\;of\;the\;control\;group}\times 100\%$$ (1)

### 2.5 Pharmacokinetic analysis

Nine Sprague Dawley rats (female, weighting 180–200 g) were randomly divided into three groups (*n* = 3), and each group was injected separately with VCR, NP/VCR, and RGD-NP/VCR at a VCR dose of 2.0 mg per kg body weight (mg (kg BW)^−1^), respectively. A sample of 500 μL venous blood from the inner canthus of each rat was collected at a predetermined time and centrifuged at 3,000 rpm for 15 min. Then, 150 μL of plasma was taken, 150 μL of methanol was added to precipitate protein, and the VCR content was measured in supernatant after centrifugation.

### 2.6 *In vivo* antitumor efficacy and safety assays

All BALB/c mice were randomly divided into four groups (*n* = 6), and each mouse was injected with 1 × 10^6^ LLC cells under the right axilla. When the tumor volume reached about 50 mm^3^, mice were treated. VCR, NP/VCR, and RGD-NP/VCR were injected intravenously into the LLC mouse model at a VCR dose of 2.0 mg (kg BW)^−1^. Mice in the control group were treated with PBS. PBS or VCR preparations were administered on days 1, 4, 8, 11, and 14, respectively. The body weight of mice and tumor volume was measured every other day. On the 17th day, all mice were slaughtered. The tumor tissues, lung, kidney, spleen, heart, and liver were separated for further investigation. The tumor volume was calculated using Eq. 2:

$${V(mm}^{3})=\frac{L\times S^{2}}{2}$$ (2)

### 2.7 Immunohistochemical and histopathological analyses

At the end of each treatment, tumor and main organ tissues were collected and cut into 5 μm slices. They were then placed on slides and dried in an incubator at 45°C. Slices were stained with hematoxylin and eosin (H&E) after being dewaxed with xylene and washed with ethanol. The immunohistochemistry protocol was as follows: 1) antigen extract and serum closure; 2) add primary antibody (anti-Ki-67, anti-Caspase-3, anti-Bcl-2, and anti-Bax); and 3) add the second antibody. H&E images were captured using a microscope, and immunohistochemical images were taken by a confocal laser scanning microscope (CLSM). Finally, the average optical density (AOD) of immunohistochemical images were measured at least thrice on different observation fields and calculated by Image Pro Plus 6.0. The tumor necrosis were counted three times on different observation fields and calculated by Image Pro Plus 6.0.

### 2.8 Western blotting

Western blot measured related proteins to confirm the antitumor mechanism of VCR preparations. For homogenate lysis, 100 mg of tumor tissue was taken, and 500 μL of RIPA (PMSF: phosphase inhibitor) was added. Centrifuge the lysate at 12,000 × g and 4°C for 10 min, the supernatant was extracted, and the protein concentration was determined using a BCA kit. SDS-PDGE electrophoresis was used to separate 100 μg of protein from each sample which was then transferred to the PDVF membrane. After completion, the PDVF film was removed and immersed in 5% skim milk for 2 h. The PDVF membrane was cut according to the molecular weight of protein and then incubated overnight at 4°C with a diluted primary antibody. After primary antibody recovery, the membrane was washed thrice with TBST, each for 10 min. The diluted secondary antibody was then added and incubated for 1 h at room temperature before being washed thrice with TBST. Finally, the PVDF membrane was uniformly dripped with ECL developer solution and photographed with a gel imager.

### 2.9 TUNEL assay

TUNEL assay kit was used to determine the apoptosis rate of LLC cells in each group by the previously described procedure (Wu et al., 2021). Briefly, the fixed tumor cells were placed in the TdT enzyme buffer at 37°C for 1 h. The slides were rinsed and counterstained with DAPI. More fluorescence expression indicated more necrosis or apoptosis induced by various VCR formulations. The mean fluorescence intensity (MFI) of fluorescence expression areas were counted at least three times on different observation fields by ImageJ software.

### 2.10 Statistical analysis

All data were presented as means ± standard deviation (SD), and all experiments were performed at least in triplicate. GraphPad Prism 8.0 was used for the statistical analysis.

## 3 Results and discussion

### 3.1 Preparation and characterization of RGD−PEG−P (LP-*co*-LC) and mPEG−P (LP-co-LC)

**FIGURE 1:**
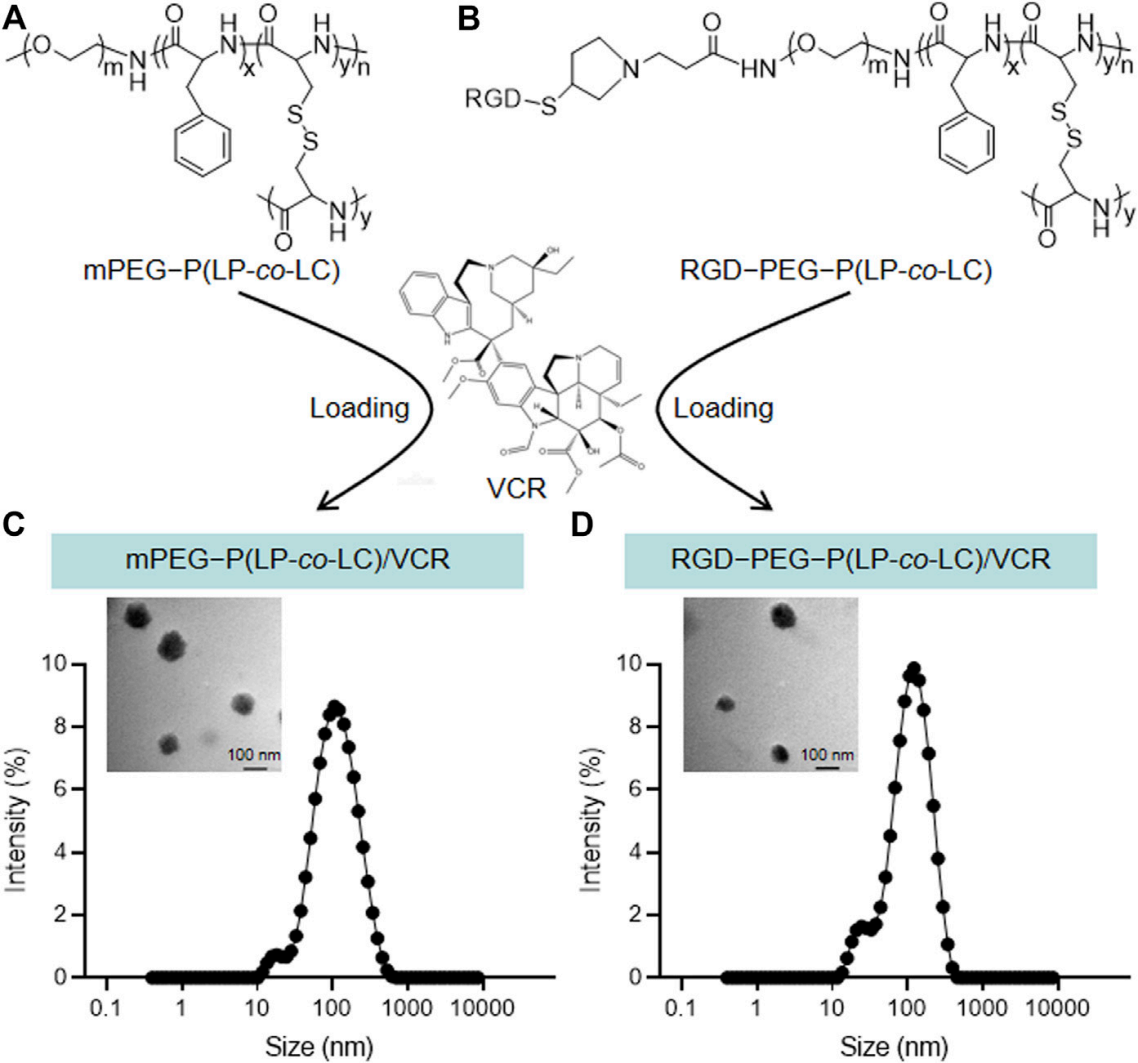
**(A,B)** The chemical structures of mPEG−P (LP-*co*-LC) and RGD−PEG−P (LP-*co*-LC). **(C,D)**The hydrodynamic diameters and TEM images (insert) of mPEG−P (LP-*co*-LC)/VCR and RGD−PEG−P (LP-*co*-LC)/VCR nanoparticles.

The chemical structures of mPEG−P (LP-co-LC) and RGD−PEG−P (LP-co-LC) were shown in Figures 1A,B. As indicated by the DLS results shown in Figures 1C,D, the mean diameters of NP/VCR and RGD-NP/VCR were 134.2 and 130.0 nm, respectively. TEM imaging showed that both nanoparticles displayed spherical morphologies [Figures 1C,D (insert)]. The diameters of NP/VCR and RGD-NP/VCR analyzed from TEM images were 108.5 ± 6.9 and 98.5 ± 11.7 nm, respectively. Both of them had suitable size distributions that can achieve passive targeting through EPR effect (Xu et al., 2017).

### 3.2 Targeted nanodrug can inhibit cell viability of LLC cells *in vitro*

**FIGURE 2:**
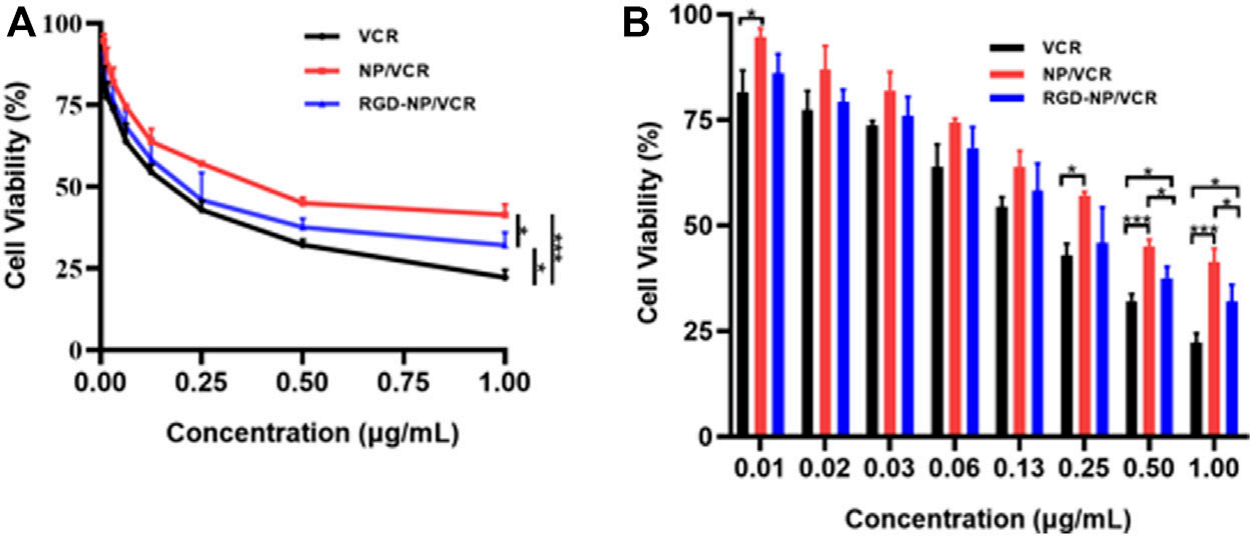
**(A,B)** The inhibition efficacies of VCR, NP/VCR, and RGD-NP/VCR on LLC cells *in vitro*. The data in each group is showed by mean ± SD (*n* = 3, NS: No significance, *p < 0.05, ***p < 0.001).

MTT assay was used to assess the cytotoxicity of VCR, NP/VCR, and RGD-NP/VCR on LLC cells. All three drugs had an inhibitory effect on LLC cells, as presented in Figures 2A,B. Three groups had a similar impact on the viability of LLC cells when the drug concentration was <0.5 μg ml^−1^. However, when drug concentration was ≥0.5 μg ml^−1^, the VCR group had the lowest cell viability. In contrast, the RGD-NP/VCR group had lower cell viability than the NP/VCR group. The IC_50_s of VCR, NP/VCR, and RGD-NP/VCR were 0.15, 0.41, and 0.22 μg ml^−1^, respectively. This is because of the relatively slow drug release from the NPs, resulting in lower cytotoxicity *in vitro* to LLC cells than in the VCR group. However, RGD-modified NPs can recognize tumor cells. Therefore, their cytotoxicity was higher than that of the NP/VCR group.

### 3.3 Targeted nanodrug has better antitumor efficacy *in vivo*

**FIGURE 3:**
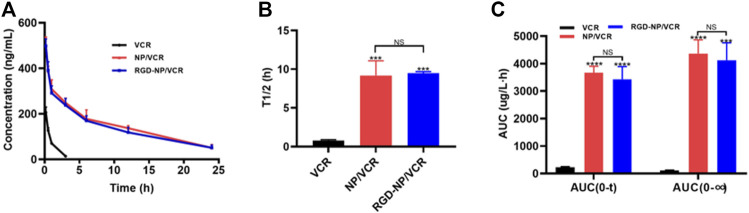
**(A)** Concentration-Time curve and **(B,C)** pharmacokinetics parameter of VCR, NP-VCR and RGD-NP/VCR. The data in each group is showed by mean ± SD (*n* = 3, NS: No significance, ***p < 0.001, ****p < 0.0001).

Pharmacokinetics is an important factor that can influence drug efficacy. The pharmacokinetics of free drugs differ significantly from drugs encapsulated in NPs, which may be due to differences in distribution characteristics of nanomaterial *in vivo* (Jeong et al., 2021). Many studies have confirmed that the bioavailability of nanodrugs is significantly improved than that of free drugs (Patel et al., 2018; Jang et al., 2019, 2020). Figure 3A indicates that the drug concentration in the RGD-NP/VCR and NP/VCR groups decreased slowly, whereas the drug concentration of the VCR group decreased rapidly. The half-lives (T_1/2_) for three VCR preparations are 0.79, 9.19, and 9.48 h, respectively. The AUC(0∼t) of VCR, NP/VCR, and RGD-NP/VCR were 228.1, 3,671.9, and 3,427.9 μg (L h)^−1^. The AUC (0∼∞) of three groups were 114.1, 4,365.3, and 4,123.6 μg (L h) ^−1^, respectively. Figures 3B,C demonstrated that the T_1/2_ and area under the curve (AUC) of drugs in the RGD-NP/VCR and NP/VCR group were higher than those in the VCR group, while there was no significant difference between RGD-NP/VCR and NP/VCR groups. These findings suggest that nanodrugs have a high bioavailability and can maintain a high blood concentration.

**FIGURE 4:**
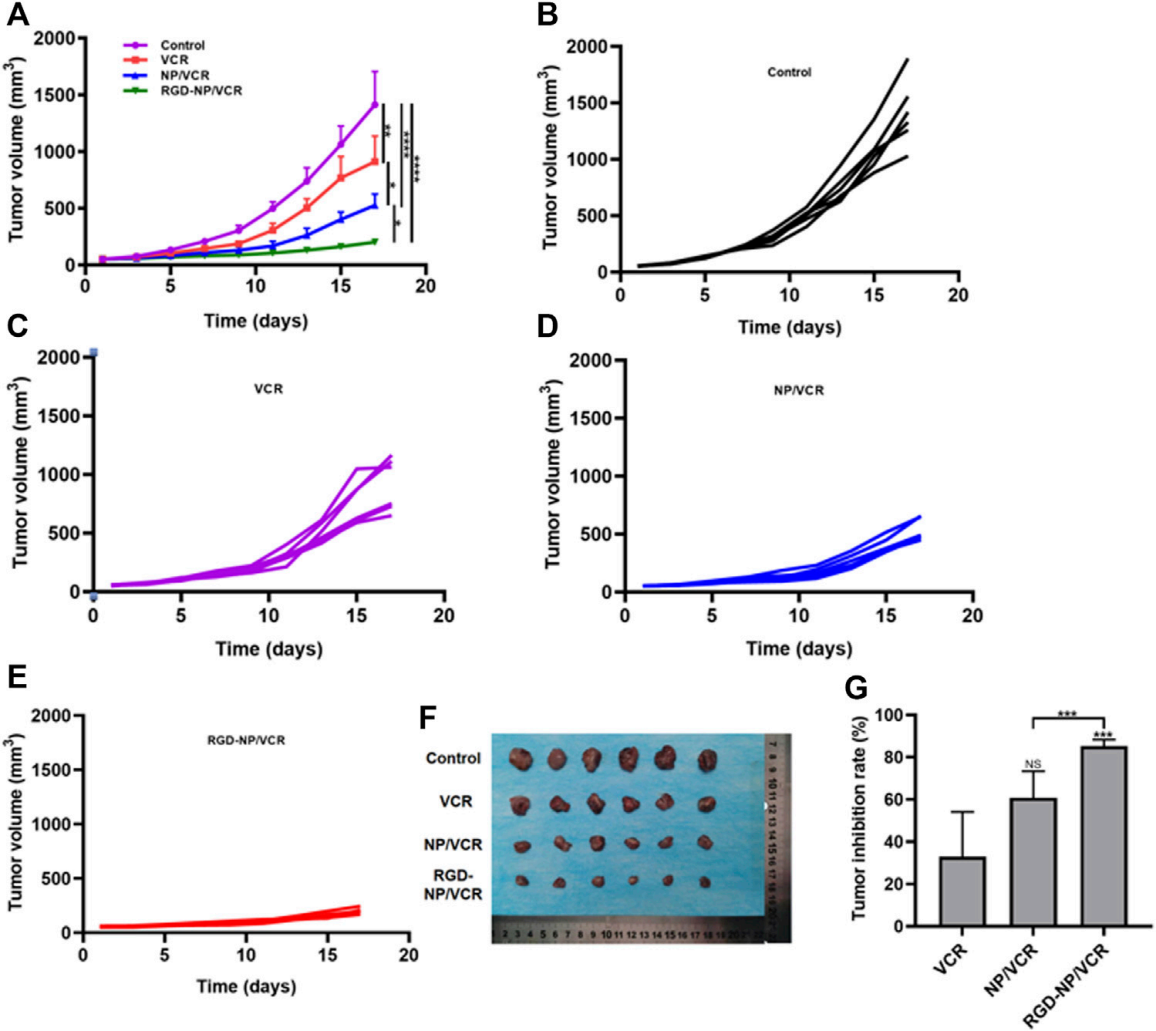
**(A)** The change in tumor volume of Control, VCR, NP/VCR, and RGD-NP/VCR group; **(B–E)** The change in tumor volume of per mouse in Control, VCR, NP/VCR, and RGD-NP/VCR group; **(F)** The tumor volume at the end of treatment; **(G)** The tumor inhibition rate of VCR, NP/VCR and RGD-NP/VCR group. The data in each group is showed by mean ± SD (*n* = 6, NS: No significance, *p < 0.05, **p < 0.01, ***p < 0.001, ****p < 0.0001).

Nanodrugs can target tumor lesions and increase drug residence time in blood, improving drug antitumor efficacy and reducing drug resistance (Su et al., 2021). Although traditional nanodrugs can address some issues, such as drug degradation and side effects, their efficacy remains very low (Cheng T.-M. et al., 2021). The RGD-modified nanocarriers can identify tumor cells and improve the antitumor efficacy of traditional nanodrugs. Figures 4A demonstrated that the RGD-NP/VCR group had the best antitumor efficacy, with a minimal change of tumor volume and the mean tumor volume increased to 203.1 mm^3^ after the treatment on the 17th day. However, the mean tumor volume in control, VCR, and NP/VCR groups were 1,414.8 mm^3^, 911.8 mm^3^, and 527.8 mm^3^, respectively, significantly higher than that in RGD-NP/VCR group. Figures 4B–E depicted the change in tumor volume of mice in each group, with the RGD-NP/VCR group growing the slowest. Figure 4F showed the tumor volume in each group and RGD-NP/VCR group was smallest. The tumor inhibition rates of the VCR, NP/VCR, and RGD-NP/VCR groups were 32.9%, 60.8%, and 85.2%, with the tumor inhibition rate in RGD-NP/VCR group being higher than other groups and statistically significant as presented in Figure 4G. The above findings demonstrated that RGD-NP/VCR effectively controlled LLC progression and had a better antitumor effect.

**FIGURE 5:**
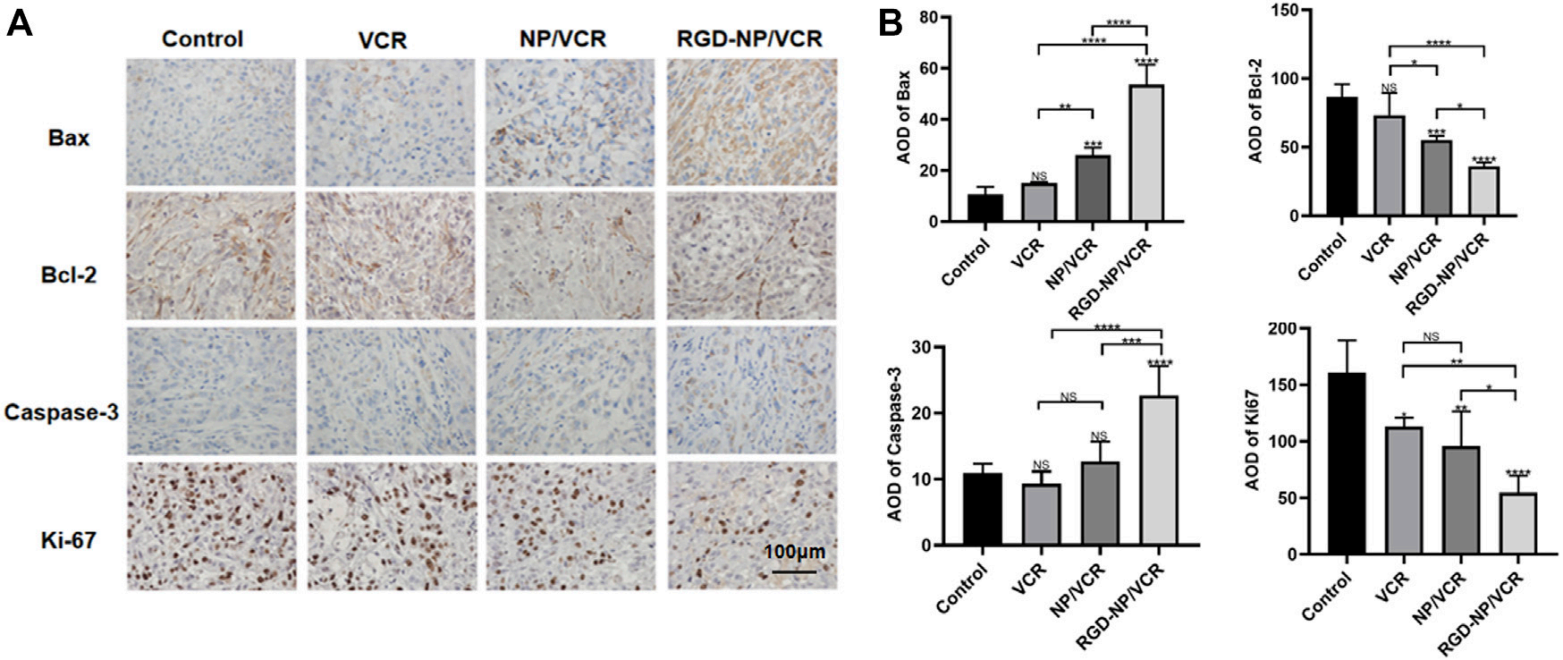
**(A)** The immunohistochemical and **(B)** its semi-quantification analysis of Control, VCR, NP/VCR, and RGD-NP/VCR group (for Bax, Bcl-2, Caspase-3, and Ki-67). The data in each group is showed by mean ± SD (*n* = 5, NS: No significance, *p < 0.05, **p < 0.01, ***p < 0.001, ****p < 0.0001).

**FIGURE 6:**
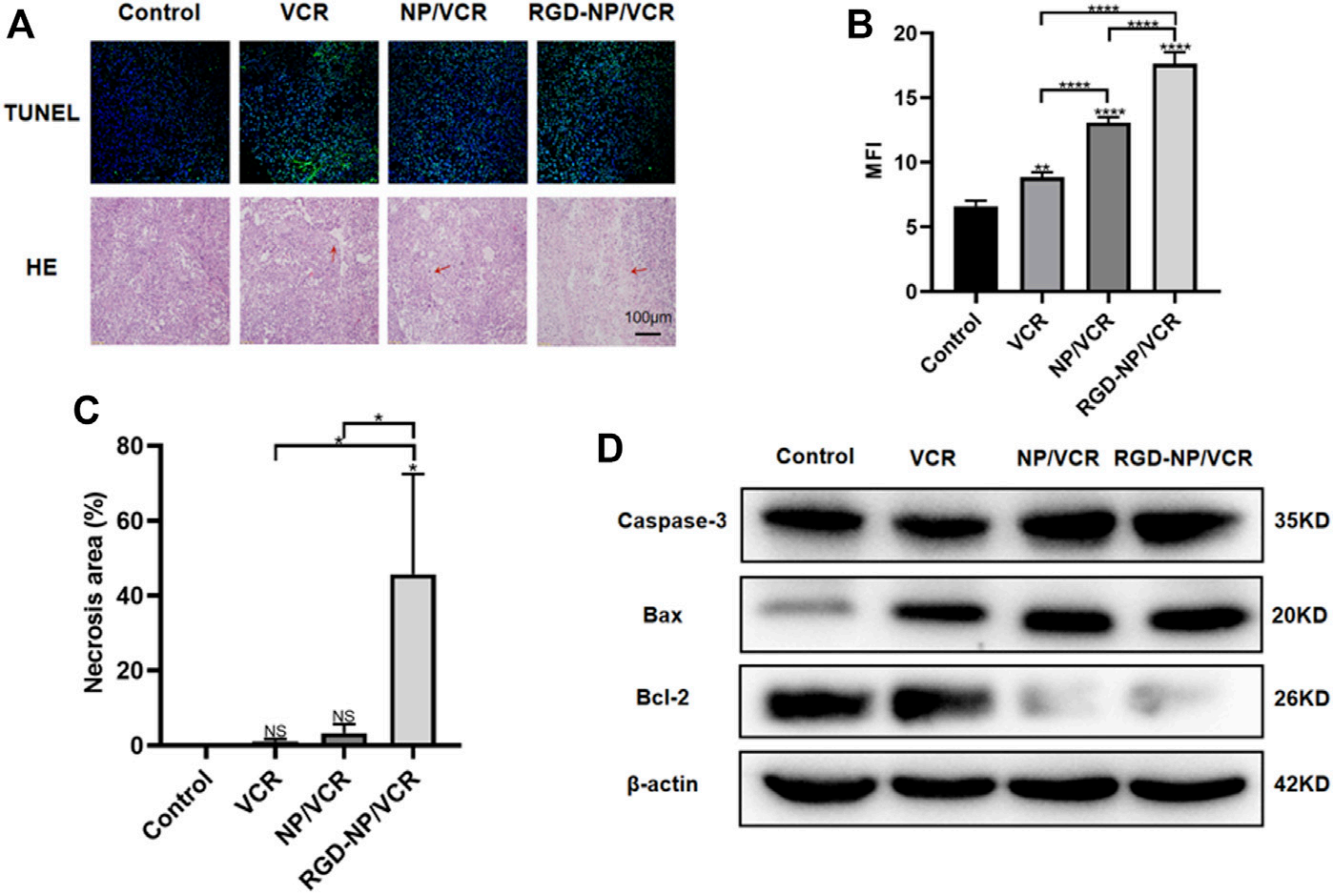
**(A,B)** The apoptosis analysis of tumor cells in each group; **(A,C)** The necrosis analysis of tumor in each group; **(D)** The analysis of apoptosis-related protein (Caspase-3, Bax, and Bcl-2) by western-blotting in each group. The data in each group is showed by mean ± SD (*n* ≥ 3, NS: No significance, *p < 0.05, **p < 0.01, ****p < 0.0001).

Exploring tumor biology at the molecular level is critical for developing new therapies (Gupta et al., 2021). Uncontrolled apoptosis is a characteristic of tumors regulated by numerous genes (Zhao et al., 2021). Studies have shown that NPs can mediate cytotoxicity through apoptosis (Mohammadinejad et al., 2019). Bax is a key protein in controlling apoptosis, and its expression is increased in cells early in cell apoptosis, whereas Bcl-2 can inhibit apoptosis and is an anti-apoptotic protein considered an oncogene (Alam et al., 2022). Decreased Bax expression and increased Bcl-2 expression can cause tumors. Caspase-3 also plays an important role in the apoptosis process, which Bcl-2 can inhibit. Ki-67 is a signature antigen for assessing cell proliferation and cell cycle, and its higher levels are associated with tumor development (Folescu et al., 2018). H&E staining, immunohistochemical, Western blot, and TUNEL assay were performed to confirm the antitumor molecular mechanism of nanodrugs. Figures 5A,B illustrated that the expressions of Ki67 and Bcl-2 in tumor tissues of RGD-NP/VCR group were lower than those of other three groups, whereas Bax and Caspase-3 were higher than other groups. Figures 6A,B presented that the RGD-NP/VCR group had higher tumor cell apoptosis than the other three groups. Figures 6A,C depicted that the necrosis area of the tumor after RGD-NP/VCR treatment was about 45.69%, while the necrosis areas of the tumor in control, VCR, and NP/VCR groups were 0.04%, 1.08%, and 3.32%, respectively. Figure 6D presented that the expression of Caspase-3 and Bax by the tumor in the RGD-NP/VCR group is higher than in the other three groups, while the Bcl-2 is lower than in the other three groups. In conclusion, these findings indicate that RGD-NP/VCR can promote apoptosis and inhibit tumor cell proliferation.

### 3.4 Safety assessment of targeted nanodrug

Mild side effects are an important factor in introducing nanodrugs into clinical use. One of the current challenges for nanodrugs is gaining a better understanding of their physical and chemical properties and demonstrating their safety through further experiments (Pasut, 2019). In the present study, we evaluated the safety of targeted nanodrug by monitoring weight change and significant organ damage in mice after administration.

**FIGURE 7:**
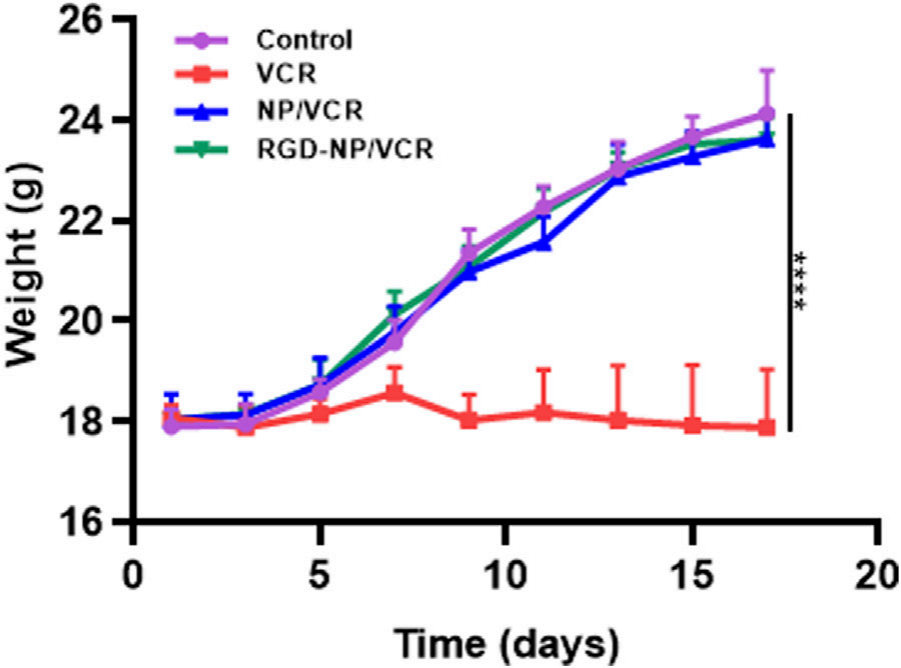
The body weight analysis of mice in each group. The data in each group is showed by mean ± SD (*n* = 6, ****p < 0.0001).

**FIGURE 8:**
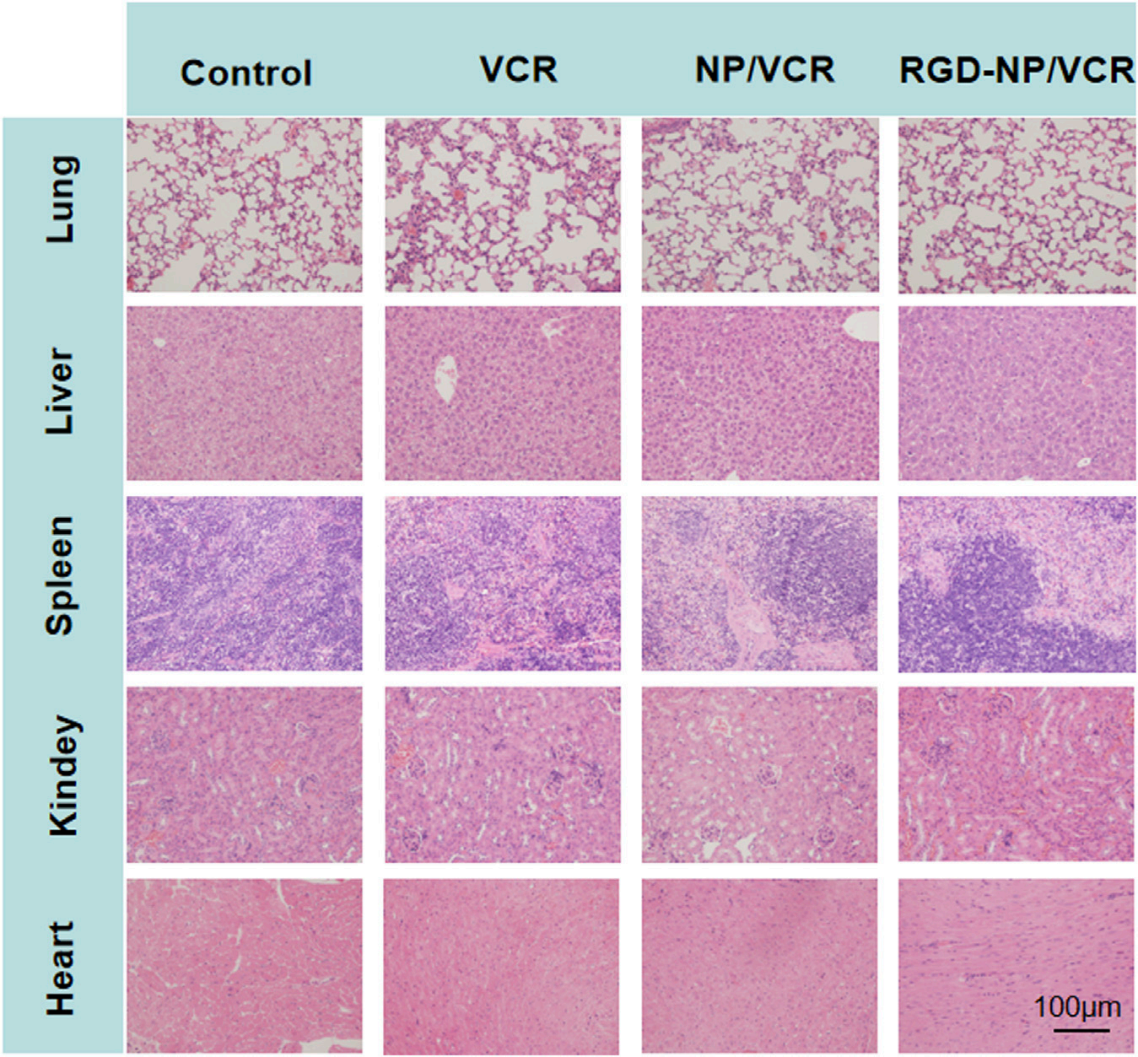
The histopathology of organs after treatment in each group (for lung, liver, spleen, kidney, and heart).

The changes in the body weight of mice during the administration were recorded to assess the safety of the nanodrugs. Figure 7 indicates that the body weight of mice in the RGD-NP/VCR and NP/VCR groups was not significantly different from that of the control group, while the weight of mice in the VCR group decreased significantly. In addition, the safety of the vital organs was evaluated. Figure 8 shows no signs of evident organ damage in any groups. The volume of hepatocytes increased slightly in the VCR group, the cytoplasm was slightly stained, and no necrosis was observed. These findings confirmed that RGD peptide-modified NPs could reduce VCR toxicity and side effects.

## 4 Conclusion

In conclusion, we prepared RGD peptide-modified NPs to encapsulate VCR and tested them in the LLC model. The RGD-NP/VCR can not only increase VCR concentration in blood and improve its antitumor efficacy, but it can also reduce side effects of VCR. Simultaneously, we investigated the antitumor mechanism of RGD-NP/VCR, and the findings confirmed that RGD-NP/VCR could inhibit tumor proliferation and promote apoptosis. The present study demonstrated that RGD-NP/VCR had high safety and efficacy, suggesting that it could be used in the clinical treatment of lung cancer.

## Data Availability

The original contributions presented in the study are included in the article/Supplementary Material, further inquiries can be directed to the corresponding author.
